# Genus* Spondias*: A Phytochemical and Pharmacological Review

**DOI:** 10.1155/2018/5382904

**Published:** 2018-02-12

**Authors:** Salma Sameh, Eman Al-Sayed, Rola M. Labib, Abdel Nasser Singab

**Affiliations:** Department of Pharmacognosy, Faculty of Pharmacy, Ain Shams University, Cairo 11566, Egypt

## Abstract

It is believed that many degenerative diseases are due to oxidative stress. In view of the limited drugs available for treating degenerative diseases, natural products represent a promising therapeutic strategy in the search for new and effective candidates for treating degenerative diseases. This review focuses on the genus* Spondias* which is widely used in traditional medicine for the treatment of many diseases.* Spondias* is a genus of flowering plants belonging to the cashew family (Anacardiaceae). This genus comprises 18 species distributed across tropical regions in the world. A variety of bioactive phytochemical constituents were isolated from different plants belonging to the genus* Spondias*. Diverse pharmacological activities were reported for the genus* Spondias* including cytotoxic, antioxidant, ulcer protective, hepatoprotective, anti-inflammatory, antiarthritic, and antidementia effects. These attributes indicate their potential to treat various degenerative diseases. The aim of this review is to draw attention to the unexplored potential of phytochemicals obtained from* Spondias* species, thereby contributing to the development of new therapeutic alternatives that may improve the health of people suffering from degenerative diseases and other health problems.

## 1. Introduction

Degenerative disease results from a continuous process based on degenerative cell changes of tissues and organs, which increasingly deteriorate over time. This might happen due to normal bodily wear or lifestyle choices such as lack of exercise or eating habits. Oxidative stress is known to be implicated in the development of degenerative diseases. An imbalance between formation and neutralization of free radicals leads to oxidative stress. These reactive species seek stability through electron pairing with biological macromolecules such as proteins, lipids, and DNA in healthy cells leading to protein and DNA damage along with lipid peroxidation [1]. These changes contribute to the development of cancer, atherosclerosis, cardiovascular diseases, aging, inflammatory diseases, and other degenerative changes. All human cells protect themselves against free radical damage by enzymes such as superoxide dismutase (SOD) and catalase, or antioxidant compounds such as ascorbic acid, tocopherol, and glutathione. Sometimes, these protective mechanisms are disrupted by various pathological processes [1]. In view of the limited drugs available for the treatment of degenerative diseases, there is an urgent need for the development of new, nontoxic, and affordable candidates for treating these diseases, especially from natural sources.

Investigation on the phytotherapy of medicinal plants that are highly valued and widely used in traditional medicine may provide efficient management for many diseases. Genus* Spondias* belongs to family Anacardiaceae which comprises 70 genera and 600 species and is endogenous mostly in the tropics and subtropics worldwide but also extends into the temperate zone. Members of this family are used in traditional medicine in the treatment of many ailments [2, 3].* Spondias* consists of 18 species, namely,* S. acida*,* S. admirabilis*,* S. chinensis* (Merr.) F. P. Metcalf (syn.* S. lakonensis* Pierre),* S. dulcis* Parkinson (syn.* S. cytherea* Sonn.),* S. expeditionaria* Hook. f.,* S. globosa* J. D. Mitch.,* S. macrocarpa* Engl.,* S. malayana* Kosterm.,* S. mombin* L. (syn.* S. aurantiaca* Schumach. & Thonn.,* S. dubia* A. Rich.,* S. graveolens* Mafad,* S. lutea* L.,* S. pseudomyrobalanus* Tussac, and* S. oghigee* G. Don),* S. novoguineensis*,* S. pinnata* (L. f.) Kurz (syn.* S. mangifera* Willd.,* S. acuminata* Roxb.),* S. purpurea* (syn.* S. myrobalanus* L.,* S. crispula* Beurl., and* S. cirouela* Tussac),* S. mexicana* S. Watson,* S. radlkoferi* Donn. Sm.,* S. tefyi* J. D. Mitch., Daly & Randrian,* S. testudinis* J. D. Mitch. and D. C. Daly,* S. tuberosa* Arruda,* S. venulosa* Engl., and* S. xerophila* Kosterm. [4, 5]. Among those species, only five species were thoroughly studied.

Members of this genus were used extensively in traditional medicine in the treatment of many ailments. Pharmacological investigation of different* Spondias* species demonstrated that these plants possess cytotoxic, antioxidant, ulcer protective, hepatoprotective, photoprotective, anti-inflammatory, antiarthritic, antidementia, antipyretic, analgesic, thrombolytic, hypoglycemic, antifertility, antihypertensive, antimicrobial, and anthelmintic activities due to the wide range of phytoconstituents that are present in this genus. Plants belonging to genus* Spondias* contain tannins, flavonoids, sterols, triterpenes, saponins, essential oils, amino acids, and polysaccharides. All the available information present in this review concerning the genus* Spondias* was compiled from official sources, namely, SciFinder, Reaxys, Google Scholar, PubMed, ScienceDirect, and Springer Link from November 2016 to August 2017. These electronic databases offer data about taxonomy, ethnopharmacology, phytochemistry, biological activities, and toxicity. The revised articles were included in this review on the basis that they are discussing the traditional uses, the phytochemical constituents, the taxonomic classification, and the pharmacological effects of the genus* Spondias*; the total number of revised articles was 73.

## 2. Taxonomic Classification

  Kingdom: Plantae
  Subkingdom: Viridiplantae
  Infrakingdom: Streptophyta
Division: Tracheophyta
  Subdivision: Spermatophytina
  Infradivision: Angiospermae
  Class: Magnoliopsida
  Superorder: Rosanae
  Order: Sapindales
  Family: Anacardiaceae
  Genus:* Spondias* [6].

## 3. Ethnopharmacology

Members of the genus* Spondias* are widely used in traditional medicine for the treatment of numerous diseases, including stomachache, diarrhoea, diabetes, dementia, anemia, dysentery, and various infections.

Considering the fruits of various species, they were used to treat many ailments. It was reported that* S. dulcis* fruits are utilized by the rural population in Bangladesh to increase eyesight and to prevent eye infections [7] while those of* S. tuberosa* are eaten by rural communities in Brazil due to their high nutritional value [8]. On the other hand, the fruits of* S. mombin* are used in Nigeria as a diuretic [9]. Powdered ripe fruits of* S. pinnata* are used in India as an antidote for poison arrows [10].

Regarding the leaves of* Spondias*, in Mexico, an infusion of the fresh leaves of* S. purpurea* is used to treat stomachache and flatulence [11]. The leaf decoction of the fresh leaves is used in the treatment of anemia, diarrhoea, dysentery, and skin infections [12–14], while in Belize, a decoction of* S. mombin* leaves is used to treat diarrhoea and dysentery as well as by populations in Nigeria, Benin, and Togo to retain good memory [3]. The aqueous extract of* S. mombin* leaves is popularly used in Brazil as an abortifacient [15]. In Southwest Nigeria, the leaves are used by traditional healers to manage diabetes mellitus [2]. They possess also antimicrobial [16] and antiviral activities [17].

The gum of* S. mombin* is used in Belize as an expectorant and to expel tapeworms [18, 19]. In India, the gum produced from* S. pinnata* is used as a a demulcent [20] and to treat bronchitis, dysentery, ulcers, diarrhoea, and skin diseases [21].

In Mexico, a decoction from the bark of* S. purpurea* is used to treat anemia, diarrhoea, dysentery, and skin infections [12–14]. In India, the bark of* S. pinnata* is used as a rubefacient for the treatment of painful joints. It is also used to treat diarrhoea and dysentery and to prevent vomiting [22]. A decoction prepared from the root bark is used to regulate menstruation and to treat gonorrhoea [23].

## 4. Phytochemical Constituents

Genus* Spondias* is rich in different classes of secondary metabolites, including phenolics, sterols, triterpenes, saponins, essential oils, amino acids, and polysaccharides (Tables 1–3).

Among the isolated phenolic compounds, geraniin and galloyl geraniin were isolated from the 80% ethanolic extract of* S. mombin* leaves and stems [24]. Galloyl glucose, rhamnetin, isorhamnetin, kaempferol, kaempferide, astragalin, isoquercetin, and quercetin dihydrate were obtained from the fruit acetone extract of* S. purpurea* [25]. Moreover, some flavonoids including rutin (quercetin 3-*O*-*β*-D-rutinoside), rhamnetin 3-*O*-*β*-D-rutinoside, and quercetin 3-*O*-[*α*-rhamnopyranosyl-(1→2)]-*α*-rhamnopyranosyl-(1→6)-*β*-glucopyranoside were isolated from the methanol extract of* S. venulosa* leaves [26]. Gallic acid and 3-caffeoyl quinic acid were isolated from the acetone extract of* S. purpurea* fruit [25]. Furthermore, methyl gallate was isolated from the methanolic extract of* S. pinnata* bark [27].

Triterpenoid compounds, including *β*-amyrin and oleanolic acid, were isolated from the methanolic extract of* S. pinnata* fruit [28]. Sterols such as stigmast-4-en-3-one, 24-methylenecycloartanone, lignoceric acid, *β*-sitosterol, and *β*-sitosterol *β*-D-glucoside were isolated from the ethanolic extract of* S. pinnata* aerial parts [29], while stigmasta-9-en-3,6,7-triol and 3-hydroxy-22-epoxystigmastane were isolated from the methanolic extract of* S. mombin* bark [30]. Ergosteryl triterpenes 1 and 2 were isolated from the chloroform/methanol extract of* S. pinnata* bark [31]. In addition, lupeol was isolated from* S. mombin* and* S. purpurea* leaves [32]. Some saponins such as echinocystic acid-3-*O*-*β*-D-galactopyranosyl (1→5)-*O*-*β*-D-xylofuranoside were isolated from the ethanolic extract of* S. mangifera* roots [33].

Various volatile oil constituents were reported from different* Spondias* species. Hydrodistillation of the leaves of* S. mombin* and* S. purpurea* led to the isolation and identification of *α*-pinene, *β*-pinene, caryophyllene, humulene, indene, and cadinene [32].

**Figure 1:**
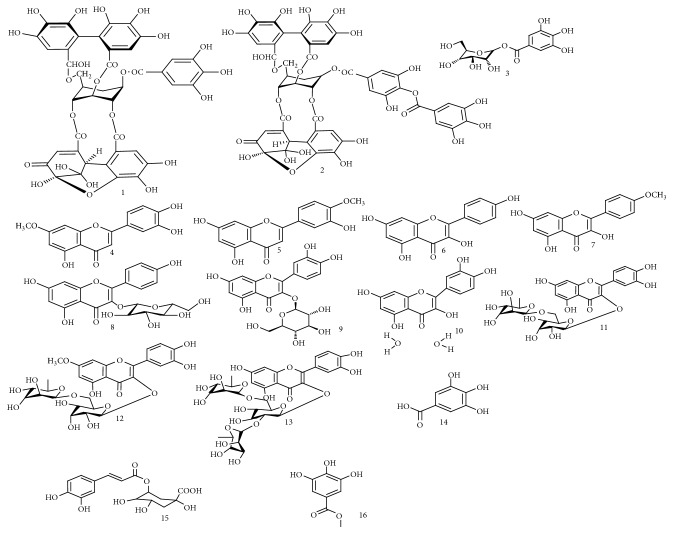
Chemical structures of phenolic compounds isolated from* Spondias* species.

**Figure 2:**
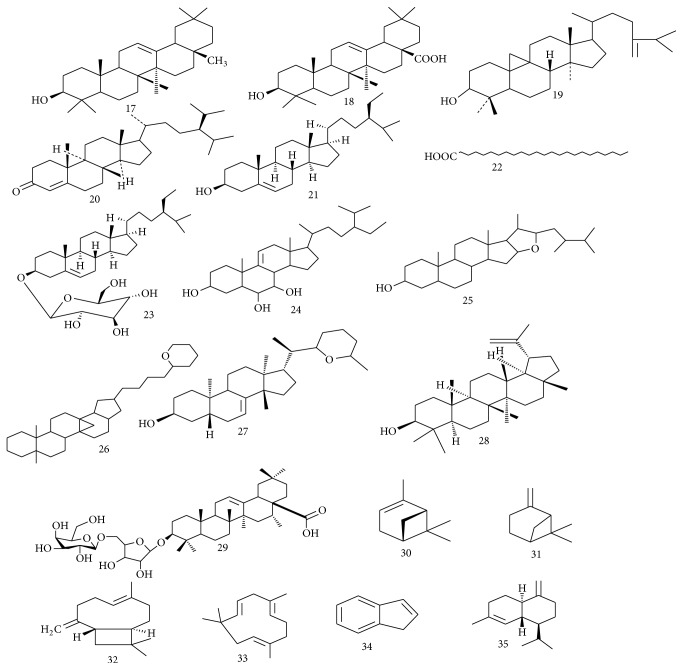
Chemical structures of sterols and terpenoids isolated from* Spondias* species.

**Figure 3:**
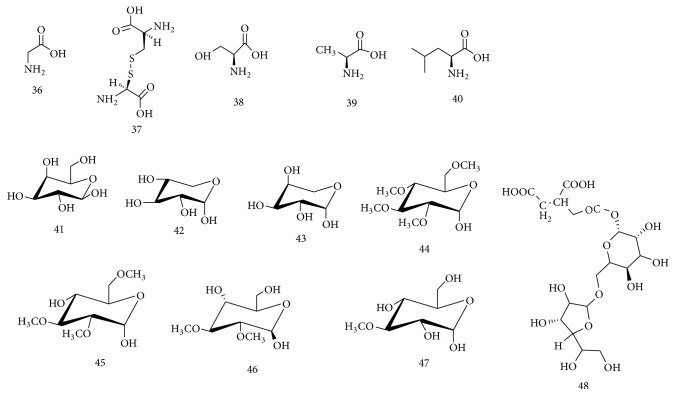
Chemical structures of amino acids and carbohydrates isolated from* Spondias* species.

The fruit of* S. pinnata* showed nutritional value and was found to be rich in several amino acids, namely, glycine, cysteine, serine, alanine, and leucine [34]. Moreover, D-galactose, D-xylose, L-arabinose, 2,3,4,6-tetra-*O*-methylglucose, 2,3,6-tri-*O*-methylglucose, 2,3-di-*O*-methylglucose, and 3-*O*-methylglucose were isolated from the aqueous extract of* S. pinnata* fruit [35]. Propan-1,2-dioic acid-3-carboxyl-*β*-D-glucopyranosyl-(6′→1′′)-*β*-D-glucofuranoside (an acid glycoside) was obtained from the ethanolic extract of* S. pinnata* fruits [36]. The chemical structures of all these compounds isolated from the genus* Spondias* are presented in Figures 1–3.

## 5. Pharmacological Effects

Different reported pharmacological activities of the genus* Spondias* are detailed below.

### 5.1. Cytotoxic Activity

Ghate et al. (2013) demonstrated that the methanolic extract of* S. pinnata* bark exhibited significant cytotoxicity on human lung adenocarcinoma (A549) and human breast adenocarcinoma (MCF-7) cell lines via inducing apoptosis.* In vitro* WST-1 cell proliferation assay was carried out; A549 cells were seeded in a 96-well culture plate at a density of 5*∗*10^4^ cells/well whereas MCF-7 cells were seeded at 1*∗*10^4^ cells/well and allowed to settle for 2 h. The cells were then treated with the methanolic extract of* S. pinnata* ranging from 0 to 200 *μ*g/ml for 48 h. The 70% methanolic extract of* S. pinnata* inhibited the growth of both A549 and MCF-7 cells in a dose-dependent manner with an IC_50_ value of 147.84 and 149.34 *μ*g/ml, respectively. Cell proliferation and viability were quantified by measuring the absorbance of the produced formazan at 460 nm using a microplate ELISA reader. The pathway of apoptosis induction may be due to an increase in Bax/Bcl-2 ratio in both cell types, which resulted in the activation of the caspase cascade, subsequently leading to cleavage of poly adeno ribose polymerase enzyme [37].

Chaudhuri et al. (2015) tested the activity of compounds isolated from the ethyl acetate fraction obtained from the bark of* S. pinnata* for their cytotoxic activity against human glioblastoma cell line (U87).* In vitro* WST-1 cytotoxicity assay was carried out; 1 × 10^4^ cells were treated with compounds isolated from the ethyl acetate fraction (1 to 30 *μ*g/ml) for 48 h in a 96-well culture plate. Two isolated compounds (gallic acid and methyl gallate) showed promising cytotoxic activities with IC_50_ of 59.28 and 8.44 *μ*g/ml, respectively [27]. Gallic acid induced cell death in promyelocytic leukemia HL-60RG cells [38]. Previous studies showed that treatment of murine tumors with methyl gallate extracted from* Moutan Cortex Radicis* enhances the antitumor effects through modulation of the function of CD4^+^CD25^+^ Treg cells.* In vitro*, methyl gallate decreased CD4^+^CD25^+^ Treg cell migration and reduced the suppressive function of effector T-cells. In tumor-bearing animals, treatment with methyl gallate delayed tumor progression and prolonged survival through inhibition of the tumor infiltration of CD4^+^CD25^+^ Treg cells [39].

### 5.2. Antioxidant Activity

Hazra et al. (2008) proved that the 70% methanolic extract of* S. mangifera* bark is a potent source of antioxidants. Total antioxidant activity was assessed* in vitro*, depending on the ability of the 70% methanolic extract to scavenge ABTS radical cation, and compared to trolox standard, the total antioxidant activity of the 70% methanolic extract was calculated from the decolorization of ABTS cation which was measured spectrophotometrically at 734 nm; the trolox equivalent antioxidant value was of 0.78 [1]. In addition,* S. mangifera* methanolic fruit extract at concentration of 5 *μ*g/ml showed 16% radical scavenging activity compared to the same concentration of vitamin C which showed only 5% radical scavenging activity [40].

Arif et al. (2016) showed that the ethanolic extract of* S. mangifera* fruits contains large amounts of phenolics, flavonoids, and acid glycosides, such as propan-1,2-dioicacid-3-carboxyl-*β*-D-glucopyranosyl-(6′→1′′)-*β*-D-glucofuranoside.* In vitro* and* in vivo* studies were conducted to test the effects of ethanolic extract and acid glycoside as antioxidants against anoxia-stress tolerance, swimming endurance, and cyclophosphamide—immune suppression. The antioxidant activity was compared to a standard drug Geriforte [36].

*An in vitro* study was carried against DPPH^*∙*^ and determined by a UV spectrophotometer at 517 nm. Aliquots of 0.05, 0.5, and 1 mg/ml of either the ethanolic extract of the acid glycoside were mixed in test tubes each containing 3 ml of methanol and 0.5 ml of 1 mM DPPH^*∙*^; ascorbic acid was used as a standard at the same concentrations, and the reaction mixture was incubated at 37°C for 30 min. The radical scavenging activity was calculated; IC_50_ was 0.32 and 0.15 mg/ml for the ethanolic extract and acid glycoside, respectively, while IC_50_ of ascorbic acid was 0.015 mg/ml. These results indicated that the ethanolic extract and the acid glycoside exhibited a significant antioxidant activity [36].

Furthermore, an* in vivo* experiment was carried out on thirty Swiss* Albino* mice which were divided into five groups of six mice each; group 1 served as the control and received vehicle alone (2% gum acacia), groups 2 and 3 were treated with 100 and 200 mg/kg/day of the ethanolic extract, group 4 was treated with 10 mg/kg/day of the acid glycoside, and group 5 was treated with 50 mg/kg/day of the standard drug Geriforte; all the groups were treated for 3 weeks; every week after 1 h of drug administration, each animal was placed in an airtight glass container of 250 ml and the time taken for appearance of generalized clonic seizures was observed (alternate limbs flexion and extension connected to loss of posture). Thereafter, the mice were removed for recovery; the time duration from the entry of the animal into the hermetic vessel to the appearance of the first convulsion was taken as the time of anoxia tolerance; the anoxia tolerance effect was increased with increasing dose and duration of treatment, indicating the significant stress relaxant activity [36].

Another* in vivo* study was carried out on 30 mice, divided into five groups of six mice each; group 1 served as the control and received vehicle alone (2% gum acacia), groups 2 and 3 were treated with 100 and 200 mg/kg/day of ethanolic extract, respectively, group 4 was treated with 10 mg/kg/day of acid glycoside, and group 5 was treated with 50 mg/kg/day of the standard drug Geriforte; all the drugs were given orally once a day for seven days; on the seventh day, 1 hour after drug administration, all the mice were made to swim in a water tank maintained at room temperature until they sank; the control group swam for 131.2 min; the ethanolic extract treated mice at doses of 100 and 200 mg/kg/day swam for 152.7 and 158.6 min, respectively, whereas the acid glycoside treated mice swam for 155.4 min. It was evident that the ethanolic extract and the acid glycoside treated mice exhibited a significant increase in physical swimming endurance time [36].

An extra* in vivo* study was carried out on 24 mice, divided into four groups of six mice each. It was observed that the administration of cyclophosphamide alone (25 mg/kg/day) produced a significant decrease in the total RBCs and leukocytes counts, whereas cyclophosphamide given along with ethanolic extract (100 mg/kg/day) and acid glycoside (10 mg/kg) conferred a good protection by increasing the haematological parameters. It was suggested, based on this study, that the ethanolic extract and acid glycoside may be coadministered with chemotherapy for the treatment of patients with severely impaired or suppressed immune system [36], as the ethanolic extract and the acid glycoside are able to reduce leukopenia and anemia induced by cyclophosphamide administration [36].

Shetty et al. (2016) conducted an* in vivo* study on Wistar rats to show the effects of combining conventional chemotherapy with* S. pinnata* bark extract to reduce chemotherapy's side effects. The rats were divided into four groups: group 1 (normal control), group 2 (received etoposide alone (i.p.) in a single dose of 60 mg/kg b.w.), group 3 (received etoposide followed by* S. pinnata* bark extract (100 mg/kg b.w.) orally once a day from 0 h to 72 h), and group 4 (received etoposide (i.p.) followed by *S. pinnata* bark extract in a dose of 200 mg/kg b.w. orally once a day from 0 h to 72 h). The results showed that animals which received chemotherapy in group 2 showed a significant decrease of GSH level in the liver and kidney tissues as compared to the control group, while treatment with* S. pinnata* bark extract after chemotherapy showed a significant increase in GSH level when compared to group 2. This study proved the protective action of the extract on the liver and kidney against chemotherapy-induced chemical stress [41].

Cabral et al. (2016) proved that the hydroethanolic extract of* S. mombin* leaves showed a significant antioxidant activity, and in an* in vitro* DPPH^*∙*^ assay, the hydroethanolic extract was tested at 60, 125, 250, and 500 *μ*g/ml and showed DPPH^*∙*^ radical scavenging activity ranging from 66% to 76% [42].

The methanol extract of* S. purpurea* fruit showed a strong free radical scavenging activity and this was deduced by carrying out an* in vitro* study to evaluate the ability of the methanol extract of* S. purpurea* fruit to sequestrate the DPPH^*∙*^ radicals; the flavonoid rutin was used as a positive control and sequestrated 90.01% of the DPPH^*∙*^ radicals at concentration 250 *μ*g/ml. while the methanol extract of* S. purpurea* fruit sequestrated 74.41% with EC_50_ of 27.11 *μ*g/ml [43]. The strong antioxidant activity of plants belonging to genus* Spondias* has been attributed mainly to their flavonoids and phenolic content [41].

### 5.3. Ulcer Protective Activity

The pathophysiology of gastric ulceration involves an imbalance between offensive and protective factors [44, 45]. Arif et al. (2008) carried out an* in vivo* study to evaluate the ulcer protective activity of* S. mangifera* methanolic bark extract. Gastric ulceration was achieved by administering different doses of indomethacin (30, 60, and 100 mg/kg) to rats orally and 100 mg/kg was found to be the most effective for producing gastric ulceration in the rats. The rats were then divided into four groups, each comprising six animals. Food and water were withdrawn 24 h and 2 h, respectively, before drug administration. Rats in group 1 received 100 mg/kg indomethacin while those in group 2 were pretreated with 100 mg/kg cimetidine. The rats in groups 3 and 4 were pretreated with 100–200 mg/kg of bark extract 1 h prior to the administration of indomethacin (100 mg/kg). The drugs were administered intragastrically. After 4 h, the animals were killed by cervical dislocation and their stomachs were removed and opened along the greater curvature. The ulcer index (UI) of each group was calculated. The groups treated with bark extract showed a marked reduction of the ulcerogenic effect of indomethacin, reducing the ulcer index from 17.7 (ulcerated control) to 8.7 and 6.7 for the groups treated with bark extracts of 100 mg/kg and 200 mg/kg, respectively. The methanolic bark extract of* S. mangifera* was thus concluded to possess a marked inhibitory effect of indomethacin-induced ulceration [46].

Sabiu et al. (2015) tested the gastroprotective and antioxidative potential of the aqueous extract of* S. mombin* leaves. Ulceration was induced in Albino rats by oral administration of indomethacin which caused a significant increase in the degree of ulceration. Pretreatment with the extract of 200 mg/kg b.w. facilitated the ulcer healing process, which was associated with a decrease in pepsin activity and an elevation in mucin levels in the gastric mucosa. Moreover,* S. mombin* leaf extract ameliorated the oxidative stress and inhibitory action of indomethacin on prostaglandin synthesis [47].

### 5.4. Hepatoprotective Activity

The ethyl acetate and methanolic extracts of* S. pinnata* stem heartwood possess a marked* in vivo* hepatoprotective effect on CCl_4_ intoxicated rats. The ethyl acetate and methanolic extracts were administered at doses of 100, 200, and 400 mg/kg, p.o., and the results showed a protective activity in a dose-dependent manner as evidenced by the significant decreases in ALT and AST to their normal levels, which was comparable to silymarin. The hepatoprotective effect in this study was attributed to the presence of flavonoids. Histopathological examination was also carried out on CCl_4_ intoxicated rats and revealed that normal hepatic architecture was retained in rats treated with* S. pinnata* extracts [48].

Hazra et al. (2013) evaluated the effect of* S. pinnata* stem bark methanol extract on iron-induced liver injury in mice. Intraperitoneal administration of iron dextran induced an iron overload and led to liver damage along with a significant increase in serum hepatic markers (ALT, AST, ALP, and bilirubin). The administration of* S. pinnata* methanol extract in doses of 50, 100, and 200 mg/kg induced a marked increase in antioxidant enzymes, along with dose-dependent inhibition of lipid peroxidation, protein oxidation, and liver fibrosis. Meanwhile, the levels of serum enzyme markers and ferritin were also reduced, suggesting that the extract is potentially useful as an iron chelating agent for iron overload diseases [49].

Chaudhuri et al. (2016) evaluated the activity of the methanolic extract of* S. pinnata* bark against iron-induced liver fibrosis and hepatocellular damage. In an iron-overloaded liver, iron reacts with cellular hydrogen peroxide to generate hydroxyl radicals which in turn initiate the propagation of various free radicals; this situation leads to oxidative stress. Two compounds (gallic acid and methyl gallate) were isolated from the ethyl acetate fraction of this extract; an* in vivo* study showed that methyl gallate exhibited better iron chelation properties than gallic acid. It was proved that methyl gallate overcomes hepatic fibrosis by ameliorating oxidative stress and sequestrating the stored iron in cells [50]. These results were in accordance with previous studies of Nabavi et al. (2013) which indicated the* in vivo* protective effect of gallic acid isolated from* Peltiphyllum peltatum* against sodium fluoride induced hepatotoxicity and oxidative stress. The results showed that gallic acid (10 and 20 mg/kg) prevented the sodium fluoride induced abnormalities in the hepatic biochemical markers; these effects were comparable to the reference drug silymarin (10 mg/kg) [51].

### 5.5. Photoprotective Activity

Ultraviolet A and ultraviolet B are known to induce skin cancer. The free radicals generated from sunlight are responsible for the degradation of essential cellular components such as DNA and proteins [43]. The UVA photoprotective activity of the ethanolic extract of* S. purpurea* fruit was assessed* in vitro* by the* trans*-resveratrol method, which indicated its marked photoprotective ability against UVA radiation [52]. Silva et al. (2016) tested the* in vitro* UVB photoprotection effect of the ethanol extract of* S. purpurea* fruit by a spectrophotometric method. The photoprotective effect was attributed to phenolic compounds in* S. purpurea* fruit extract having the ability to absorb the solar radiation, to scavenge free radicals, and to decrease the harmful effects of the sun [43].

### 5.6. Anti-Inflammatory Activity

The hydroethanolic extract of* S. mombin* leaves showed a significant anti-inflammatory activity in a carrageenan-induced peritonitis model in mice. Carrageenan induced neutrophil migration to the peritoneal cavity and typical signs of acute inflammation including vasodilation, edema, and leukocyte infiltration. It was evident from this study that* S. mombin* leaf extract (100, 200, 300, and 500 mg/kg) reduced the leukocyte influx to the peritoneal cavity of the treated animals [42].

da Silva Siqueira et al. (2016) showed that phenolic compounds were responsible for the anti-inflammatory activity exhibited by* S. tuberosa* leaves hydroethanolic extract. Furthermore, an* in vivo* study was conducted on Swiss Albino mice, where dexamethasone was used as a standard anti-inflammatory drug and carrageenan was used to induce hind paw edema. The extract (125, 250, and 500 mg/kg) induced significant amelioration of the inflammatory response induced by carrageenan, a marked reduction in the number of leukocytes in the peritoneal cavity, and a significant decrease in myeloperoxidase activity [53].

### 5.7. Antiarthritic Activity

Nitric oxide plays an important role in various inflammatory processes. However, sustained levels of production of this radical are directly toxic to tissues and contribute to the vascular collapse associated with septic shock, whereas chronic expression of nitric oxide radical is associated with various degenerative diseases, including carcinomas and inflammatory conditions such as juvenile diabetes, multiple sclerosis, arthritis, and ulcerative colitis. The toxicity of NO increases greatly when it reacts with a superoxide radical, forming the highly reactive peroxynitrite anion (ONOO-). Hazra et al. (2008) proved that the methanolic extract of* S. pinnata* inhibits nitrite formation* in vitro* by directly competing with oxygen in the reaction with nitric oxide. The results revealed that IC_50_ of the methanolic extract (tested at 200 *μ*g/ml) was 716.32 *μ*g/ml which was lower than that of the reference compound gallic acid (IC_50_ = 876.24 *μ*g/ml). The scavenging percentages were 22.3 and 15.8% for* S. pinnata* and gallic acid, respectively. This study proved that the extract exhibited more potent peroxynitrite radical scavenging activity than the standard gallic acid [1].

### 5.8. Learning and Memory

The ability to acquire knowledge and to retain this acquired knowledge can be defined as learning and memory. Several conditions such as aging and stress may lead to the impairment of learning. It has been shown that aging may lead to various neurodegenerative processes including memory loss, dementia, and Alzheimer's disease [54]. Asuquo et al. (2013) proved that the aqueous extract of* S. mombin* leaves (400, 800 mg/kg b.w.) enhanced the learning and memory capabilities of Wister rats due to structural changes observed in the cerebrum. Improved learning and memory have been also linked to structural changes of the limbic system [55]. The aqueous extract may have also positively affected the biosynthesis of neurotransmitters, such as acetylcholine, noradrenaline, dopamine, and 5-HT that are involved in learning and memory mechanisms [56, 57]. Ishola et al. (2017) investigated the* in vivo* protective effect of the hydroethanolic leaf extract of* S. mombin* (50, 100, or 200 mg/kg, p.o.) and proved the protective effect against scopolamine-induced cognitive dysfunction and memory deficit that could be attributed to the extract antioxidant properties [58].

### 5.9. Analgesic and Antipyretic Activities

Panda et al. (2009) tested the analgesic activity of the ethanolic extract of* S. pinnata* bark. The analgesic activity was evaluated using acetic acid, formalin test, and hot plate model. The extract showed a dose-dependent analgesic effect (50–100 mg/kg, p.o.) in the acetic acid test, comparable to the effect of acetyl salicylic acid. Terpenoids, flavonoids, and tannins were responsible for the analgesic activity [59]. Panda et al. (2014) also evaluated the antipyretic activity of* S. pinnata* bark ethanol extract (200 and 400 mg/kg, p.o.). Pyrexia was induced in Albino rats by brewer's yeast. The extract showed a significant reduction in pyrexia, which continued for 5 hours after drug administration [60].

### 5.10. Thrombolytic Activity

Manik et al. (2013) showed that both ethyl acetate and aqueous extracts of* S. pinnata* fruit at the concentration of 10 mg/ml have a significant thrombolytic activity compared to streptokinase as a standard substance [61]. Kamal et al. (2015) proved that the ethanolic extract of* S. pinnata* (1 mg/ml) leaves has a membrane stabilizing activity for human RBCs in hypotonic solution-induced hemolysis. In case of heat-induced hemolysis,* S. pinnata* extracts produced marked inhibition of hemolysis [62]. Uddin et al. (2016) demonstrated the possible thrombolytic and membrane stabilizing activities of the ethanolic extract of* S. pinnata* aerial parts and its different fractions. The ethyl acetate fraction exerted the highest thrombolytic activity and membrane stabilizing activity [63].

### 5.11. Hypoglycemic Activity

The hypoglycemic activity was tested using different extracts of the genus* Spondias.* The leaves of* S. mombin* were tested* in vitro* by Fred-Jaiyesimi et al. (2009) for their hypoglycemic activity. A new compound, 3*β*-olean-12-en-3-yl (9*Z*)-hexadec-9-enoate, isolated from the diethyl ether fraction of the methanolic extract of* S. mombin* leaves, showed an *α*-amylase inhibitory activity similar to the activity of acarbose. The methanolic leaf extract and the isolated new compound decreased postprandial hyperglycemia. The methanolic extract (250 mg/ml) showed 39% inhibition of the *α-*amylase activity, while the diethyl ether fraction (70 mg/ml) showed 73% inhibition and the isolated compound (20 mg/ml) exhibited 57% *α*-amylase inhibition [2].

Mondal and Dash (2009) showed a promising hypoglycemic effect of the methanolic bark extract of* S. pinnata*, which was comparable to glibenclamide. The test was carried out* in vivo*, and the methanolic extract was administered at a dose of 300 mg/kg to rats. After 30 min of treatment, rats were loaded orally with glucose (2 g/kg, p.o.). Blood samples were collected before and at 30, 90, and 150 min intervals after glucose administration, the methanol extract was found to reduce blood glucose level by 63.12%, and the results were found to be comparable to glibenclamide [64].

Acharyya et al. (2010) tested the hypoglycemic activity of both the methanolic and the aqueous extracts of* S. pinnata* roots* in vivo* using oral glucose tolerance test and indicated a significant decrease in blood glucose levels after four hours of treatment as compared to glibenclamide [65].

### 5.12. Antifertility Activity

Asuquo et al. (2013) carried out a study on adult female Wister rats to determine the effect of the ethanolic extract of* S. mombin* leaves on anterior pituitary, ovary, uterus, and serum sex hormones. The animals received the ethanolic extract at dose levels of 250, 350, and 500 mg/kg b.w. The results showed a significant decrease in the weight of pituitary, ovary, and uterus of the treated animals, along with a significant reduction in FSH, LH, estradiol, and progesterone levels. Therefore, this study concluded that the extract showed antifertility activity and can be used as a contraceptive [66].

### 5.13. Antihypertensive Activity

Das and De (2013) tested the* in vitro* antihypertensive activity of the aqueous extract of* S. pinnata* fruit (20 *μ*g/ml). The angiotensin-converting-enzyme inhibitory activity was assayed using ACE from rabbit lung and N-hippuryl-L-histidyl-L-leucine as a substrate. This showed 50% inhibition of ACE enzyme [67].

### 5.14. Antimicrobial Activity

Arif et al. (2008) tested the* in vitro* antibacterial activity of the methanolic and the aqueous extracts of* S. pinnata* bark by cup plate diffusion method at the concentrations of 50, 100, and 150 mg. The activity was tested against* Escherichia coli*,* Salmonella* Typhimurium, and* Vibrio cholerae* and compared with penicillin and streptomycin as standard drugs. The methanolic extract showed a good antibacterial activity against Gram +ve and Gram −ve bacteria, while the aqueous extract showed only a mild antibacterial activity. The resin of* S. pinnata* also showed an antibacterial activity against* Bacillus subtilis* [46].

The 80% ethanolic extract of* S. pinnata* fruits showed a strong antibacterial activity against both Gram +ve and Gram −ve bacteria. The antimicrobial activity was tested by disc diffusion method; standard discs of kanamycin (30 *μ*g/disc) and blank discs were used as positive and negative controls, respectively [68].

Tapan et al. (2014) isolated two new ergosteryl triterpenes (SP-40, SP-60) from* S. pinnata* bark and tested their antipseudomonal activity by agar disc diffusion method against a moderately resistant strain of* Pseudomonas aeruginosa* MTCC 8158. The tested organism was completely resistant to ampicillin and tetracycline at concentrations of 10 and 30 *μ*g/disc, respectively, while exhibiting an inhibition zone of 15 mm against streptomycin at 100 *μ*g/disc concentration. SP-40 exhibited an inhibition zone of 20 mm, which was better than streptomycin at comparable concentrations. SP-60, however, did not show any antimicrobial activity against this organism up to a concentration of 200 *μ*g/disc. The MIC values of SP-40, thus, were estimated to be between 25 and 12.5 *μ*g/disc [31].

Olugbuyiro et al. (2013) isolated two new phytosterols: stigmasta-9-en-3,6,7-triol and 3-hydroxy-22-epoxystigmastane from the methanolic extract of* S. mombin* stem bark. Both compounds exhibited a marked antimycobacterial activity with 93% inhibition against* Mycobacterium tuberculosis* by a fluorometric microplate Alamar Blue Assay [30].

Furthermore, the methanolic fruit extract of* S. purpurea* showed a strong antimicrobial activity against* E. coli* and* P. aeruginosa* using the disc diffusion method [43]. Islam et al. (2013) observed similar results when evaluating the antimicrobial activity of* S. dulcis* fruit [69].

### 5.15. Anthelmintic Activity

The ethanolic and acetone extracts of* S*.* pinnata* bark were tested for anthelmintic activity. Florido and Cortiguerra (2003) and Kumar et al. (2012) proved that the ethanolic extract with a concentration range of 50 mg/ml and 100 mg/ml showed more potent activity than the acetone extract [70, 71]. The bark of* S. pinnata* was shown to exhibit an anthelmintic activity against Indian earthworms due to different glycosides present in the bark. Mondal et al. (2010) tested the anthelmintic activity of the chloroform extract of the bark of* S. pinnata* (10, 15, and 20 mg/ml) and showed promising effects [72].

### 5.16. Diuretic and Laxative Activity

Mondal et al. (2009) showed that the administration of chloroform and the methanol extracts of* S. pinnata* bark (300 mg/kg) to Wister Albino rats produced significant diuretic and laxative activities as compared to reference standards furosemide and agar [73].

### 5.17. Antiepileptic and Antipsychotic Activity

Ayoka et al. (2006) conducted an* in vivo* study using the methanolic and ethanolic extracts of* S. mombin* leaves and showed promising antiepileptic and antipsychotic effects. They also tested the effects of aqueous, methanolic, and ethanolic extracts of* S. mombin* on hexobarbital-induced sleep in mice. Animals given hexobarbitone (100 mg/kg i.p.) showed loss of writhing reflex within five minutes of administration. The administration of the aqueous extract (100 mg/kg) decreased the latency of sleep significantly and was more potent in increasing hexobarbitone-induced sleeping time in mice. The methanolic extract did not alter the latency of sleep, whereas it increased the latency time at doses of 12.5 and 50 mg/kg. The three extracts produced a dose-dependent prolongation of hexobarbitone-induced sleeping time in mice [74].

## 6. Toxicity

It was evident that oral administration of aqueous, methanolic, and ethanolic extracts of* S. mombin* leaves (≤5 g/kg) did not produce any toxic effects in mice and rats. Intraperitoneal administration of the aqueous extract (≤200 mg/kg) also did not produce any toxic effects; however, the ethanolic and methanolic extracts (>100 mg/kg) produced toxic symptoms. Lethal effects were observed in mice and rats with the three extracts at the dose of 3.2 g/kg administered i.p. LD_50_ in mice for ethanolic extracts was 480 mg/kg while it was 1.1 g/kg for the methanol extract and 1.36 g/kg for the aqueous extract. Also, LD_50_ in rats for the ethanolic, methanolic, and aqueous extracts was 620 mg/kg, 1.08, and 1.42 g/kg, respectively. The LD_50_ determination of the extracts was carried out in a 48 h continuous observation [74].

Mondal and Dash (2009) tested the acute* in vivo* toxicity of chloroform, methanol, and aqueous extracts of* S. pinnata* bark. The animals were divided into different groups of six animals each. The control group received 1% Tween-80 in normal saline (2 ml/kg, p.o.). The other groups received 100, 200, 300, 600, 800, 1000, 2000, and 3000 mg/kg of the tested extracts, respectively, in a similar manner. Immediately after dosing, the animals were observed continuously for the first 4 h for any behavioural changes. They were then kept under observation for up to 14 days after drug administration to find out the mortality rate if any. It was found that the chloroform and methanol extract induced sedation, diuresis, and purgation at all tested doses. However, there was no mortality in any of the extracts at the tested doses till the end of the observation period [64].

Based on these results, it can be concluded that the aqueous extract is the safest one among the tested extracts. Furthermore, the aqueous extract showed a variety of pharmacological activities using different* in vitro* and* in vivo* models which could validate its ethnopharmacological use. This evidence of use and the absence of toxicity can provide an important basis for the development of herbal medicines from the aqueous extract of different* Spondias* species.

## 7. Conclusion

Presently, there is an increased demand worldwide for the use of natural remedies. Herbal medicines could be used as a complementary or alternative medicine to synthetic drugs, and this requires more laboratory investigations on their pharmacological activities. Many degenerative diseases are associated with oxidative stress. There is an increased demand worldwide for nontoxic, easily accessible, and affordable antioxidants of natural origin. Plants belonging to the genus* Spondias* were widely used in traditional medicine due to their beneficial therapeutic effects. This is attributed to their diverse bioactive phytoconstituents like phenolics and flavonoids which possess marked antioxidant activity and thus are capable of preventing many degenerative diseases. The present review provides a comprehensive understanding of the chemistry and pharmacology of* Spondias* species, which may help in the discovery of new candidates for the treatment of various degenerative diseases and health problems.

## Figures and Tables

**Table 1:** Phenolic compounds and their occurrence in *Spondias* species.

Number	Compound	Species	Part used (type of extract)	Reference(s)
	*(A) Tannins and Pseudotannins*			
1	Geraniin	*S. mombin*	Leaves and stems (80% EtOH)	[24]
2	Galloyl geraniin	*S. mombin*	Leaves and stems (80% EtOH)	[24]
3	Galloyl glucose	*S. purpurea*	Fruits (acetone)	[25]

	*(B) Flavonoids*			
4	Rhamnetin	*S. purpurea*	Fruits (acetone)	[25]
5	Isorhamnetin	*S. purpurea*	Fruits (acetone)	[25]
6	Kaempferol	*S. purpurea*	Fruits (acetone)	[25]
7	Kaempferide	*S. purpurea*	Fruits (acetone)	[25]
8	Astragalin	*S. purpurea*	Fruits (acetone)	[25]
9	Isoquercetin	*S. purpurea*	Fruits (acetone)	[25]
10	Quercetin dihydrate	*S. purpurea*	Fruits (acetone)	[25]
11	Rutin (quercetin 3-*O*-*β*-D-rutinoside)	*S. venulosa*	Leaves (80% MeOH)	[26]
12	Rhamnetin 3-*O*-*β*-D-rutinoside	*S. venulosa*	Leaves (80% MeOH)	[26]
13	Quercetin 3-*O*-[*α*-rhamnopyranosyl-(1→2)]-*α*-rhamnopyranosyl-(1→6)-*β*-glucopyranoside	*S. venulosa*	Leaves (80% MeOH)	[26]

	*(C) Phenolic acid derivatives*			
14	Gallic acid	*S. purpurea*	Fruits (acetone)	[25]
15	3-Caffeoyl quinic acid	*S. purpurea*	Fruits (acetone)	[25]
16	Methyl gallate	*S. pinnata*	Bark (70% MeOH)	[27]

**Table 2:** Sterols and terpenoids and their occurrence in *Spondias *species.

Number	Compound	Species	Part used (type of extract)	Reference(s)
17	*β*-Amyrin	*S. pinnata*	Fruit (MeOH)	[28]
18	Oleanolic acid	*S. pinnata*	Fruit (MeOH)	[28]
19	24-Methylenecycloartanone	*S. pinnata*	Aerial parts (EtOH)	[29]
20	Stigmast-4-en-3-one	*S. pinnata*	Aerial parts (EtOH)	[29]
21	*β*-Sitosterol	*S. pinnata*	Aerial parts (EtOH)	[29]
22	Lignoceric acid	*S. pinnata*	Aerial parts (EtOH)	[29]
23	*β*-Sitosterol *β*-D-glucoside	*S. pinnata*	Aerial parts (EtOH)	[29]
24	Stigmasta-9-en-3,6,7-triol	*S. mombin*	Bark (MeOH)	[30]
25	3-Hydroxy-22-epoxystigmastane	*S. mombin*	Bark (MeOH)	[30]
26	Ergosteryl triterpene 1	*S. pinnata*	Bark (CHCl_3_/MeOH)	[31]
27	Ergosteryl triterpene 2	*S. pinnata*	Bark (CHCl_3_/MeOH)	[31]
28	Lupeol	*S. mombin, S. purpurea*	Leaves (hydrodistillation)	[32]
29	Echinocystic acid-3-*O*-*β*-D-galactopyranosyl (1→5)-*O*-*β*-D-xylofuranoside	*S. pinnata*	Roots (EtOH)	[33]
30	*α*-Pinene	*S. mombin, S. purpurea*	Leaves (hydrodistillation)	[32]
31	*β*-Pinene	*S. mombin, S. purpurea*	Leaves (hydrodistillation)	[32]
32	Caryophyllene	*S. mombin, S. purpurea*	Leaves (hydrodistillation)	[32]
33	Humulene	*S. mombin, S. purpurea*	Leaves (hydrodistillation)	[32]
34	Indene	*S. mombin, S. purpurea*	Leaves (hydrodistillation)	[32]
35	Cadinene	*S. mombin, S. purpurea*	Leaves (hydrodistillation)	[32]

**Table 3:** Amino acids and carbohydrates and their occurrence in *Spondias *species.

Number	Compound	Species	Part used (type of extract)	Reference(s)
36	Glycine	*S. pinnata*	Fruits	[34]
37	Cysteine	*S. pinnata*	Fruits	[34]
38	Serine	*S. pinnata*	Fruits	[34]
39	Alanine	*S. pinnata*	Fruits	[34]
40	Leucine	*S. pinnata*	Fruits	[34]
41	D-Galactose	*S. pinnata*	Fruits (aqueous)	[35]
42	D-Xylose	*S. pinnata*	Fruits (aqueous)	[35]
43	L-Arabinose	*S. pinnata*	Fruits (aqueous)	[35]
44	2,3,4,6-Tetra-*O*-methylglucose	*S. pinnata*	Fruits (aqueous)	[35]
45	2,3,6-Tri-*O*-methylglucose	*S. pinnata*	Fruits (aqueous)	[35]
46	2,3-Di-*O*-methylglucose	*S. pinnata*	Fruits (aqueous)	[35]
47	3-*O*-Methylglucose	*S. pinnata*	Fruits (aqueous)	[35]
48	Propan-1,2-dioic acid-3-carboxyl-*β*-D-glucopyranosyl-(6′→1′′)-*β*-D-glucofuranoside	*S. pinnata*	Fruits (EtOH)	[36]
